# Integrating m6A Regulators-Mediated Methylation Modification Models and Tumor Immune Microenvironment Characterization in Caucasian and Chinese Low-Grade Gliomas

**DOI:** 10.3389/fcell.2021.725764

**Published:** 2021-11-25

**Authors:** Wangrui Liu, Chuanyu Li, Yuhao Wu, Wenhao Xu, Shuxian Chen, Hailiang Zhang, Haineng Huang, Shuai Zhao, Jian Wang

**Affiliations:** ^1^Department of Transplantation, Xinhua Hospital Affiliated to Shanghai Jiao Tong University School of Medicine, Shanghai, China; ^2^Department of Neurosurgery, Affiliated Hospital of Youjiang Medical University for Nationalities, Guangxi, China; ^3^School of Medicine, Tongji University, Shanghai, China; ^4^Department of Urology, Fudan University Shanghai Cancer Center, Shanghai Medical College, Fudan University, Shanghai, China; ^5^Department of Oncology, Xinhua Hospital Affiliated to Shanghai Jiao Tong University School of Medicine, Shanghai, China

**Keywords:** m6A, low-grade glioma, tumor environment, immunotherapy, prognosis, RNA modificatio

## Abstract

**Background:** As an important epigenetic modification, m6A methylation plays an essential role in post-transcriptional regulation and tumor development. It is urgently needed to comprehensively and rigorously explore the prognostic value of m6A regulators and its association with tumor microenvironment (TME) infiltration characterization of low-grade glioma (LGG).

**Methods:** Based on the expression of 20 m6A regulatory factors, we comprehensively evaluated the m6A modification patterns of LGG after unsupervised clustering. Subsequent analysis of the differences between these groups was performed to obtain m6A-related genes, then consistent clustering was conducted to generate m6AgeneclusterA and m6AgeneclusterB. A Random Forest and machining learning algorithms were used to reduce dimensionality, identify TME characteristics and predict responses for LGG patients receiving immunotherapies.

**Results:** Evident differential m6A regulators were found in mutation, CNV and TME characteristics of LGG. Based on TCGA and CGGA databases, we identified that m6A regulators clusterA could significantly predict better prognosis (*p* = 0.00016) which enriched in mTOR signaling pathway, basal transcription factors, accompanied by elevated immune cells infiltration, and decreased IDH and TP53 mutations. We also investigated the distribution of differential genes in m6A regulators clusters which was closely associated with tumor immune microenvironment through three independent cohort comparisons. Next, we established m6Ascore based on previous m6A model, which accurately predicts outcomes in 1089 LGG patients (*p* < 0.0001) from discovering cohort and 497 LGG patients from testing cohort. Significant TME characteristics, including genome heterogeneity, abidance of immune cells, and clinicopathologic parameters have been found between m6Ascore groups. Importantly, LGG patients with high m6Ascore are confronted with significantly decreased responses to chemotherapies, but benefit more from immunotherapies.

**Conclusion:** In conclusion, this study first demonstrates that m6A modification is crucial participant in tumorigenesis and TME infiltration characterization of LGG based on large-scale cohorts. The m6Ascore provides useful and accurately predict of prognosis and clinical responses to chemotherapy, immunotherapy and therapeutic strategy development for LGG patients.

## Background

Low-grade glioma (LGG) is a progressive, invasive, and chronic central nervous system disease. LGGs are a group of heterogeneous neuroepithelial tumors that originate from the supporting glial cells in the central nervous system (Rueda et al., 2011; Duffau and Taillandier, 2015; Wesseling and Capper, 2018). Although the tumor progression is relative slow, and these tumors may undergo malignant transformation, leading to the development of high-grade gliomas. At present, the average survival time of LGG patients is generally less than 10 years (Wessels et al., 2003). The available treatment options for LGGs remain controversial and require further investigation. Regardless of the classic therapy strategy of resection followed by chemotherapy or novel developed personalized treatments based on specific molecular markers of tumors (Wessels et al., 2003; Duffau, 2007; Martino et al., 2009; Louis et al., 2014), the primary purpose is to extend the overall survival (OS) of the patients. Therefore, the development of a model that can evaluate the survival and prognosis of patients is urgently needed to assist clinicians in the effective treatment of LGG patients.

Although some cases showed remarkable clinical efficacy (Dunin-Horkawicz et al., 2006; Alarcón et al., 2015; Patil et al., 2016), most of patients did not benefit from immunotherapy, suggesting there are still unmet clinical needs in LGG treatment (Zhao et al., 2017). The tumor microenvironment (TME) is composed of cancer cells, stromal cells (cancer-associated fibroblasts and macrophages), and recruited immune cells that influence the development and progression of cancer. Tumor cells interact with the TME to modify the purity of the tumor, causing changes in various biological behaviors, such as the induction of immune tolerance, tumor proliferation, and angiogenesis and the inhibition of apoptosis (Wang et al., 2017). Determining the degree of TME cell infiltration and tumor purity to predict the blocking effect of immune checkpoint inhibitors is an essential step to improve the success rate of existing immunotherapies and develop new treatment strategies (Wang et al., 2017, 2018).

The methyltransferases (m6A “writers”), demethylases (m6A “erasers”), and m6A “reader” proteins coordinate in the process of m6A modification. m6A RNA methylation is considered to be the most important and abundant form of internal modification in eukaryotic cells (Granier et al., 2016; Cui et al., 2017; Pinello et al., 2018; Luo et al., 2021). According to previous reports, m6A regulatory factors play a vital role in RNA splicing, export, stability and translation et (Topalian et al., 2012; Helmy et al., 2013; Chen et al., 2021; Zheng et al., 2021). Recent studies have shown that m6A is associated with glioma (Topalian et al., 2012; Tu et al., 2020; Du et al., 2021), but its specific roles and mechanisms are still unknown.

In recent years, several studies have revealed that the TME is associated with m6A. It has been reported that m6A enhances the anti-cancer response of tumor-infiltrating CD8+ T cells, improves the therapeutic effect of anti-PDL1 receptor blockers (Wood et al., 2014; Wang et al., 2019). In addition, previous studies demonstrated that the abnormal expression of m6A regulators induces tumor proliferation and metastasis (Quail and Joyce, 2013). However, because of technical limitations, most p studies were limited to one or two m6A modulators and cell types, while the anti-tumor effect is characterized by several tumor suppressors interacting in a highly cooperative network. In summary, elucidating the mechanisms underlying TME cell infiltration mediated by multiple m6A regulatory factors will help to our further understanding of TME immune regulation. Furthermore, the potential role of m6A methylation modification in LGG remains unclear. Based on the expression of 21 m6A regulatory factors, this study comprehensively evaluated m6A modification patterns in LGG samples from The Cancer Genome Atlas (TCGA) and Chinese Glioma Genome Atlas (CGGA) databases and compared these results with data from the Affiliated Hospital of YouJiang Medical University for Nationalities (AHYMUN) for verification. Surprisingly, we found that evaluating the m6A modification pattern within a single LGG could predict patient prognosis and tumor progression. We also developed a comprehensive scoring system to quantify the m6A modification pattern in each LGG patient and enable the accurate prediction of specific prognosis and immunotherapy efficacy. Significantly, we first demonstrated the function of m6A modification in facilitating LGG progression and provides promising target for prognostic or therapeutic prediction of LGG.

## Materials and Methods

### Data

TCGA data: download the mRNA expression profile data and sample CNV (Copy number variation) information of low-grade glioma samples from https://xenabrowser.net/datapages/, clinical information using R package cgdsr, mutation data using R package TCGAbiolinks (Colaprico et al., 2016). In addition, we downloaded the expression profiles of two sets of low-grade glioma samples from http://www.cgga.org.cn/. Specific data information are shown in Table 1.

**TABLE 1:** Sample information form.

Data set	TCGA	CGGA
IDH		
Wild-type	33	144
Mutant	91	439
NA	375	0
Age		
≥60	68	28
<60	431	596
OS.Status		
Deceased	125	291
Living	374	309

### Data Preprocessing

In order to maintain data consistency, we used the Bioconductor -sva 1 package of R software (version 4.0.0) (Chan, 2018) to perform batch correction on low-level glioma transcriptome data downloaded from TCGA and CGGA databases.

### Unsupervised Clustering Using 20 m6A Genes

Extract the expressions of 21 regulators from the TCGA and CGGA datasets to identify the different m6A modification patterns mediated by the m6A regulators, of which the expression of *IGF2BP1* is not detected in the CGGA dataset, so the last 20 extracted regulators the expression of the child. The 20 m6A regulatory factors include 8 writers (*METTL3*, *METTL14*, *RBM15*, *RBM15B*, *WTAP*, *KIAA1429*, *CBLL1*, *ZC3H13*), 2 erasers (*ALKBH5*, *FTO*), and 10 readers (*YTHDC1*, *YTHDC2*, *YTHDF1*, *YTHDF2*, *YTHDF3*, *HNRNPA2B1*, *HNRNPC*, *FMR1*, *LRPPRC*, *ELAVL1*). Using unsupervised cluster analysis, according to the expression of 20 m6A regulatory factors, identify different m6A modification patterns, and classify patients for further analysis. A consistent clustering algorithm is used to determine the number of clusters and their stability. We used the ConsensusClusterPlus (Wilkerson and Hayes, 2010) package for the operation. The distance used for clustering is the Euclidean distance, and repeated 1,000 times to ensure the stability of the classification.

### Gene Set Variation Analysis and Single Sample Gene Set Enrichment Analysis

In order to study the difference of m6A modification patterns in biological processes, we used R package GSVA to perform GSVA enrichment analysis. GSVA is a non-parametric, unsupervised method that is mainly used to estimate changes in pathways and biological process activity in samples. Download the c2.cp.kegg.v6.2 gene set from the MSigDB database^1^ for running GSVA analysis.

In order to evaluate the ratio and difference of 24 immune cells in different m6A regulators cluster, we used ssGSEA (single sample gene set enrichment analysis) analysis in the R package GSVA to obtain the infiltration ratio of 24 immune cells. Then use the Wilcox test to compare the differences between different m6A regulators cluster samples, and perform cox regression analysis on the different cells to compare the prognostic differences.

### Identify the Differentially Expressed Genes Between Different m6A Regulators Cluster

Based on the expression of 20 m6A genes, we divided the low-grade gliomas in the TCGA and CGGA databases into two categories, and used the R package limma (Ritchie et al., 2015) to determine the DEGs between different groups. The significance standard for determining the difference gene is set as *p*-value < 0.05 (after BH correction), and the difference multiple is greater than 2 times or less than 0.5 times.

### m6asocre Calculation

For the differential genes obtained in the previous analysis, use the random forest method to remove redundant genes, and then perform survival analysis on the remaining genes, filter out genes that are less related to survival (*p*-value < 0.05 is considered to be related to survival), and then use cox The regression model divides genes into two categories (coefficient is positive or negative). Refer to the Gene-gene interaction (GGI) score 4 to calculate m6Ascore using the following formula.


m6Ascore=scale(∑X-∑Y)

X is the expression value of the gene set where Cox coefficient is positive, and Y is the expression value of the gene set where Cox coefficient is negative. Using the median of m6Ascore, the samples were then divided into m6Ascore-high and m6Ascore-low, and the correlation between these two types of samples and prognosis was further analyzed.

### Correlation Between m6Ascore and Other Biological Processes

Mariathasan et al. (2018) constructed a set of genes to store genes related to certain biological processes, including immune checkpoints; antigen processing and presentation; *EMT1*, *EMT2*, *EMT3* and other epithelial-mesenchymal transitions (*EMT*) Markers; DNA damage repair; mismatch repair; nucleotide excision repair and other pathways. We conducted a Pearson correlation analysis on m6Ascore and these biological processes, and further revealed the connection between m6Ascore and some related biological pathways.

### Copy Number Variation Analysis

The GISTIC method was used to detect the common copy number change area in all samples based on SNP6 CopyNumber segment data. The parameters of the GISTIC method are set as: Q ≤ 0.05 as the change significance standard; when determining the peak interval, the confidence level is 0.95. The analysis is performed by the corresponding MutSigCV module in the online analysis tool GenePattern^2^ developed by Broad Research Institute.

### Tumor Immune Dysfunction and Exclusion Forecast and IC50 Estimate

Further, we use the R package pRRophetic to estimate the IC50 value of drugs (Cisplatin, Gemcitabine) based on the expression profile, and compare the differences in IC50 between m6Ascore high and low samples.

Researchers from Harvard developed the TIDE (tumor immune dysfunction and exclusion) tool^3^ (Jiang et al., 2018) to evaluate the clinical effects of immune checkpoint suppression therapy, with higher tumor TIDE prediction scores and poorer immune checkpoint suppression the treatment effect is related, and it has a poor prognosis. Because of the five types of tumors with reliable tumor immune dysfunction and rejection characteristics that researchers can calculate, only melanoma has publicly available patient data on anti-PD1 or anti-CTLA4 treatment, so the prognosis of immune checkpoint treatment in this analysis the prediction is done using TIDE score.

### Statistical Analysis

In the significance analysis between various scores, the Wilcox test was used to compare the differences between the two groups of samples. In the drawing display, ns means *p* > 0.05, ^∗^ means *p* < = 0.05, ^∗∗^: means *p* < = 0.01, ^∗∗∗^ means *p* < = 0.001, ^****^ means *p* < = 0.0001. The Kaplan-Meier method was used to generate a survival curve for prognostic analysis, and the log-rank test was used to determine the significance of the difference. Receiver operating characteristic (ROC) curve is used to evaluate m6Ascore’s prediction of immunotherapy, and the area under the curve (AUC) is quantified using R package pROC. When displaying mutation maps, use the R package maftools to present the mutation landscape of patients with m6Ascore high subtype and low subtype. The R package RCircos was used to plot the chromosome distribution of 21 m6A regulatory factors in 23 pairs of chromosomes.

## Results

Our research was divided into five steps. First, we downloaded three datasets from the TCGA and CCGA databases and performed m6A gene expression, mutation, and CNV analysis based on the collected data. Followed by unsupervised clustering of m6A genes, we performed GSVA enrichment, differential gene expression, mutation profiles, and clinical features analyses. Next, we verified and consistently clustered the m6A-related genes. We then identified characteristic genes through Random Forest and Cox regression analysis. Finally, we established the m6Ascore and identified its relationship with TME characteristics. The flow chart of study process is summarized in Supplementary Figure 1.

### Genetic Variation in m6A Regulatory Factors of Low-Grade Glioma From the Cancer Genome Atlas and Chinese Glioma Genome Atlas Databases

**FIGURE 1:**
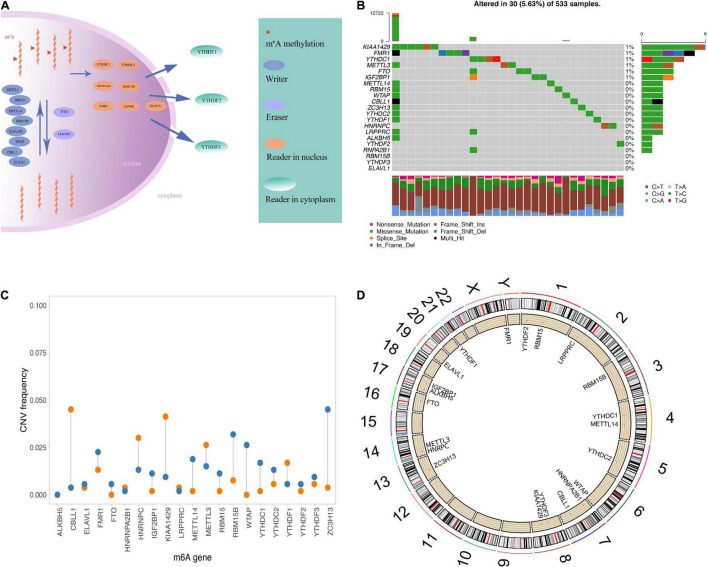
Genetic variation of m6A regulatory factors. **(A)** Summary of the dynamic reversible process of m6A RNA methylation mediated by regulators (“writers,” “erasers” and “readers”) and their potential biological functions for RNA. **(B)** The distribution of m6A gene mutations and the distribution of different mutation types. **(C)** The frequency of CNV occurrence of m6A gene, blue indicates deletion, orange indicates amplification. **(D)** m6A gene is in Position on chromosome.

A total of 21 m6A regulators analyzed in this study included 8 writers, 2 erasers, and 11 readers. Because there was no control sample in the TCGA data, it was not possible to compare the expression of these m6A regulatory factors between LGG and control samples. Figure 1A displays the dynamic process of m6A RNA methylation mediated by all known regulators.

In the experiments mentioned above, we observed that the expression of m6A regulatory factors was generally higher in the worse prognosis group. To explore the relationship between these regulatory factors and the prognosis of LGG, we compared TCGA and CGGA samples using the median expression of 20 regulatory factors, which was divided into two groups for Kaplan–Meier analysis (Supplementary Figure 2).

Then, we summarized the frequency of the copy number variations and somatic mutations of the 20 m6A regulatory factors in the LGG samples. Only a few mutations in the m6A regulators were observed in these samples, including *KIAA1429, FMR1, YTHDC1, METTL3, FTO, IGF2BO1*, and *METTL14* (Figure 1B). The CNV was generally different among the 21 regulatory factors, some of which showed copy number amplification, and the deletion frequency of genes was high, such as *FTO*, *RBM15B*, and *ZC3H13* (Figure 1C). In addition, we showed the position of the m6A regulator on the chromosome (Figure 1D). Overall, we analyzed genetic background and variation of 21 m6A regulators of LGG.

### Unsupervised Clustering of m6A Genes in 1,089 Low-Grade Glioma Samples

**FIGURE 2:**
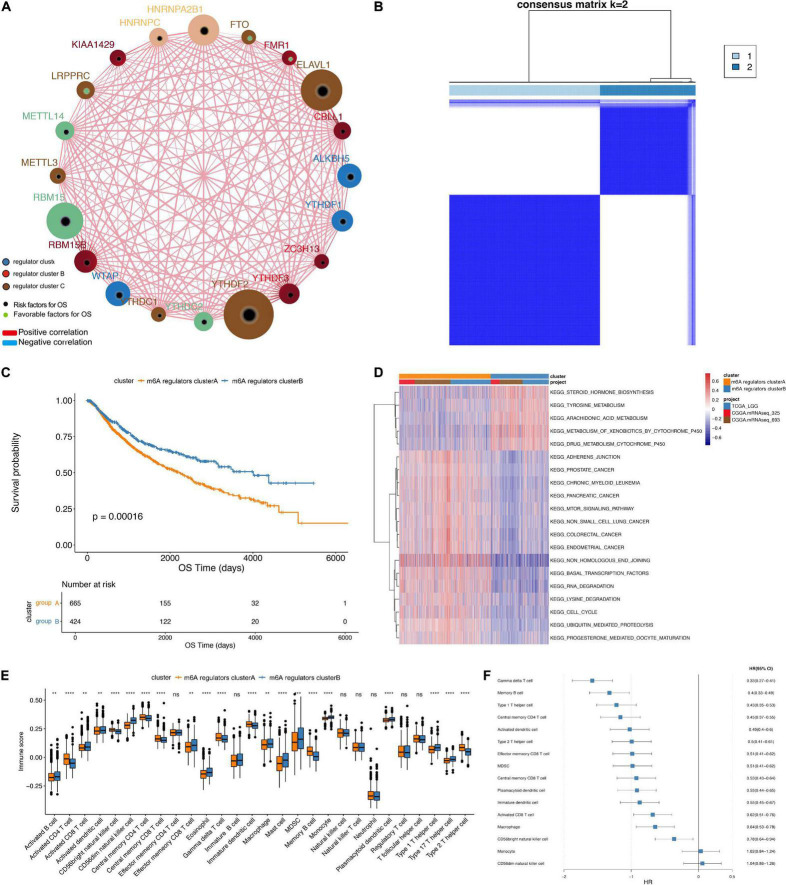
Unsupervised clustering of m6A genes in low-grade glioma samples. **(A)** Interaction between m6A genes. The size of the circle indicates the impact of each gene on survival prediction, and the larger the expression, the more relevant the prognosis. In the circle the green dots in the circle indicate prognostic protective factors, and the black dots in the circle indicate prognostic risk factors. The lines connecting genes show their interactions. The negative correlations are marked in blue and positive correlations in red. Gene clusters ABC are marked in blue, respectively, color, red and brown. **(B)** Consistent clustering of m6A genes. **(C)** Kaplan-Meier curve showing significant survival differences in two m6A regulators clusters. **(D)** GSVA enrichment analysis, showing the biological pathways with different m6A regulators clusters Activation state. Heat map is used to visualize these biological processes, red means activation, blue means inhibition. **(E)** The distribution of immune infiltration of 22 immune cells in 2 m6A regulators clusters (**p < 0.05, ****p < 0.001). **(F)** Differential cell prognosis analysis.

As *IGF2BP1* was not expressed in the CGGA data set, we used the gene expression profile data of 20 m6A regulators and the survival data in TCGA and CGGA samples to perform m6A gene consistency clustering and m6A gene single factor Cox regression analysis. The m6A regulatory network shown in Figure 2A describes the interactions between m6A regulatory factors, showing their correlation and predictive risk for OS. The impact of m6A regulators on the correlation in the interaction and the prognosis of LGG patients were shown in Supplementary Table 1. These results suggested that the interactions between m6A regulatory factors of different functional categories play a crucial role in the establishment of m6A modification patterns of LGG. Next, we determined the expression of 20 m6A regulators in LGG samples from the TCGA and CGGA databases and then used the R package ConsensusClusterPlus to perform consistent clustering. Two significant subgroups, m6A regulators clusterA and m6A regulators clusterB were indicated (Figure 2B). Patients in m6A regulators clusterB showed significantly prolonged survival compared with m6A regulators clusterA patients (*p* = 0.00016, Figure 2C).

### Functional Annotations and Tumor Microenvironment Infiltration Characterization Between m6A Regulators Clusters

Based on three datasets, we performed GSVA enrichment analysis to explore the differences in the biological behavior of the regulatory factors in two m6A modification subgroup, m6A regulators clusterA and m6A regulators clusterB. As shown in Figure 2D, m6A regulators clusterA was significantly enriched in biological processes, such as adhesion junctions, mTOR signaling, basal transcription factors and cancer-specific pathways. Nevertheless, m6A regulators clusterB was significantly enriched in differentiated processes, including steroid hormone biosynthesis, tyrosine metabolism, arachidonic acid metabolism and etc.

Furthermore, we performed ssGSEA analysis to obtain the proportion of immune cells infiltrations, like B memory cells, activated dendritic cells, M0 macrophages (Figure 2E). The results revealed significantly different distribution of immune cells abundance in the two subgroups. Next, we depict the results of univariate Cox regression analysis of immune cells with different proportions between the two m6A regulators clusters (Figure 2F). Proportion of immune cells infiltrated in different subgroups of LGG were listed in Table 2.

**TABLE 2:** Proportion of immune cells in LGG.

Immune cells	*p*-value	HR	Low 95%CI	High 95%CI
Activated CD8 T cell	2.42E-06	0.623838	0.512717	0.759042
Activated dendritic cell	4.28E-12	0.49214	0.402692	0.601457
CD56bright natural killer cell	0.011365	0.777254	0.639479	0.944712
CD56dim natural killer cell	0.685581	1.040787	0.857652	1.263026
Central memory CD4 T cell	7.37E-15	0.447318	0.365237	0.547845
Central memory CD8 T cell	3.04E-10	0.527204	0.431959	0.643451
Effector memory CD8 T cell	1.91E-11	0.505114	0.413792	0.61659
Gamma delta T cell	2.82E-25	0.334964	0.272504	0.411739
Immature dendritic cell	2.82E-09	0.545516	0.44667	0.666235
Macrophage	8.03E-06	0.639708	0.525777	0.778326
MDSC	2.36E-11	0.506285	0.41465	0.61817
Memory B cell	1.44E-18	0.399784	0.325899	0.490421
Monocyte	0.814155	1.023477	0.843431	1.241957
Plasmacytoid dendritic cell	3.59E-10	0.530418	0.435062	0.646675
Type 1 T helper cell	3.88E-16	0.42936	0.350297	0.526267
Type 2 T helper cell	1.98E-11	0.502187	0.410632	0.614155

**FIGURE 3:**
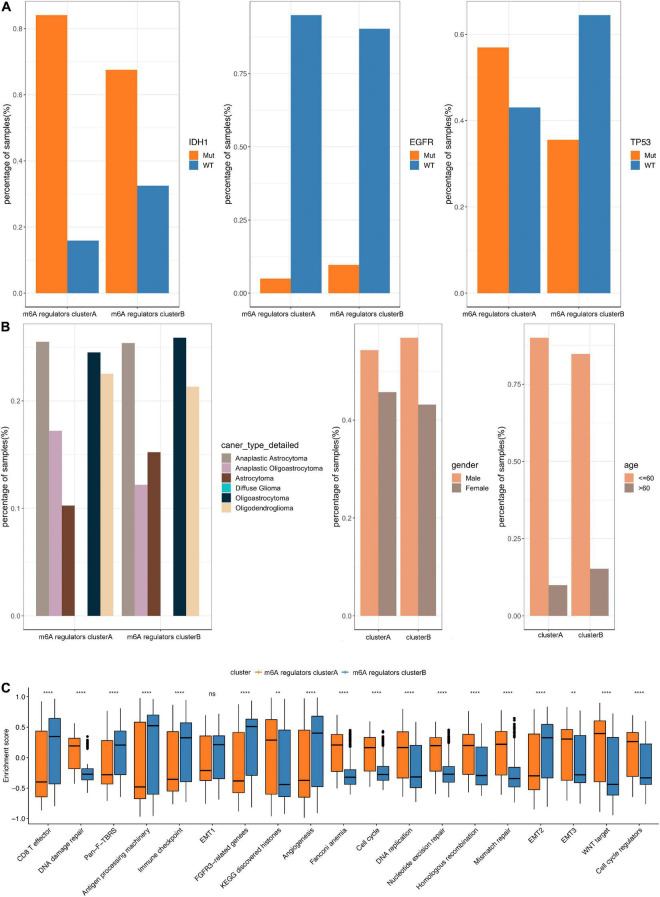
Comparative analysis between m6A 4 regulators cluster in the TCGA dataset. **(A)** The distribution of IDH1, EGFR, TP53 mutations in the 2 m6A regulators clusters. **(B)** The distribution of cancer type, gender, and age in m6A regulators cluster. **(C)** The enrichment scores of different m6A regulators cluster groups difference (**p < 0.05, ****p < 0.001).

**FIGURE 4:**
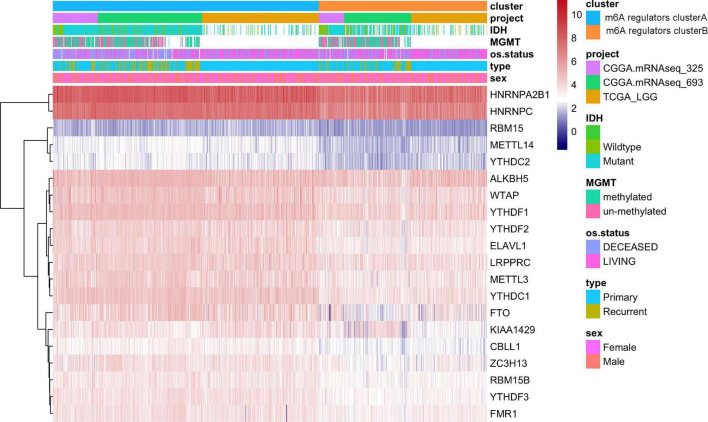
The expression of m6A regulatory factors in m6A regulators cluster.

In TCGA dataset, we found that the *IDH1* (chi-square test, *p* = 2.31e-05) and *TP53* (chi-square test, *p* = 4.47e-06) mutation were significantly more frequently in m6A regulators clusterB, while *EGFR* (chi-square test, *p* = 0.065) mutation was relatively decreased compared with m6A regulators clusterA subgroup (Figure 3A). In terms of clinical characteristics, such as cancer types, gender and age, there was no significant difference in the two subgroups (Figure 3B). Subsequently, we performed GSVA analysis, and the enrichment scores in the m6A regulators cluster groups were significantly different (Figure 3C). Most m6A regulators were highly expressed in m6A regulators clusterB. Taken together, there are evident distributions of tumor microenvironment infiltration characterization, genetic variation and prognosis between m6A regulators clusters of LGG (Figure 4).

### Differential Expressed m6A-Related Genes and Constructions of m6Agenecluster

**FIGURE 5:**
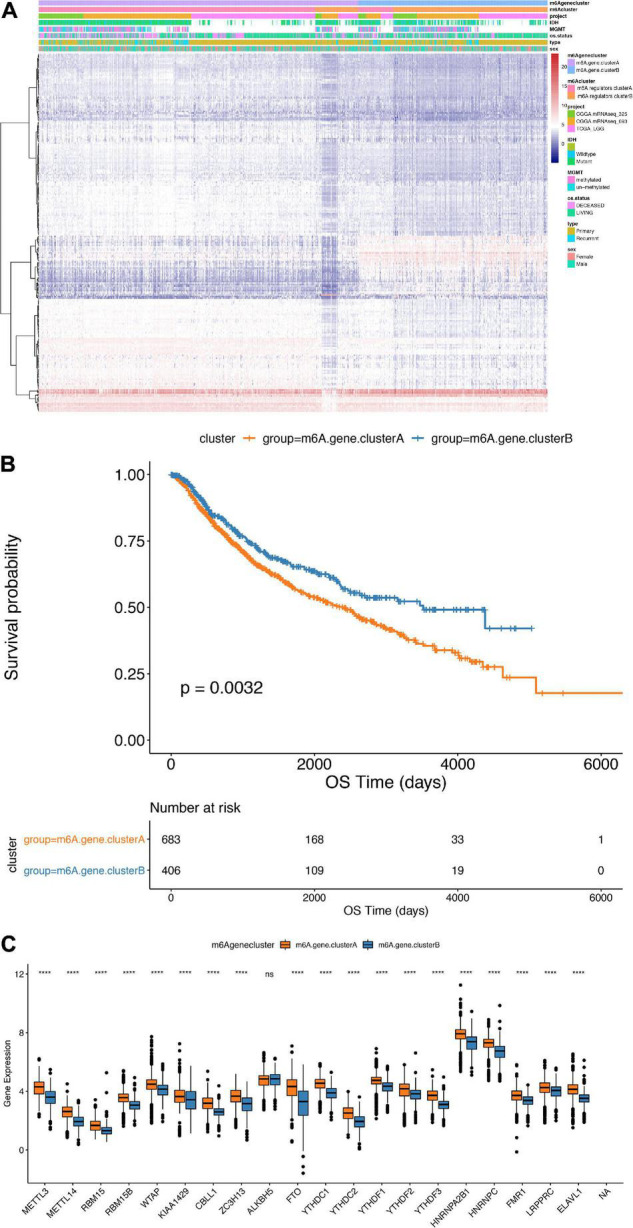
Comparison between m6Agenecluster. **(A)** Unsupervised clustering of m6A phenotype-related genes in low-grade glioma samples. The samples are divided into different genomic subtypes, called m6AgeneclusterA and m6AgeneclusterB. **(B)** Kaplan-Meier curve indicates that m6A modifies the genome table type has an obvious relationship with overall survival rate. **(C)** Expression of 20 m6A genes in 2 gene clusters. The upper and lower ends of the box indicate the interquartile range of values. The line in the box indicates the median value, and the black dots indicate outliers. The *t*-test is used to test the statistical differences between gene clusters (****p < 0.001).

To further study the potential biological behavior of the regulators in m6A regulators clusters, we exploited the “limma” R package and identified m6A phenotype-related DEGs and the clusterProfiler package to perform KEGG enrichment analysis on the DEGs. Next, we identified 238 DEGs, which were significantly enriched in the cell cycle pathway. Furthermore, we performed an unsupervised cluster analysis of the obtained m6A phenotype-related genes to group patients according to different genomic subtypes. Then, we obtained two different clusters of m6A-modified genome phenotypes, m6AgeneclusterA and m6AgeneclusterB (Figure 5A). It suggested that m6AgeneclusterA subgroup had significantly poor survival in 1,089 LGG patients (Figure 5B). In addition, the expression of most m6A regulatory factors in m6AgeneclusterA was significantly higher than that in m6AgeneclusterB (Figure 5C).

### Establishment of m6Ascore and Its Association With Tumor Microenvironment Characterization in Chinese Glioma Genome Atlas Database

**FIGURE 6:**
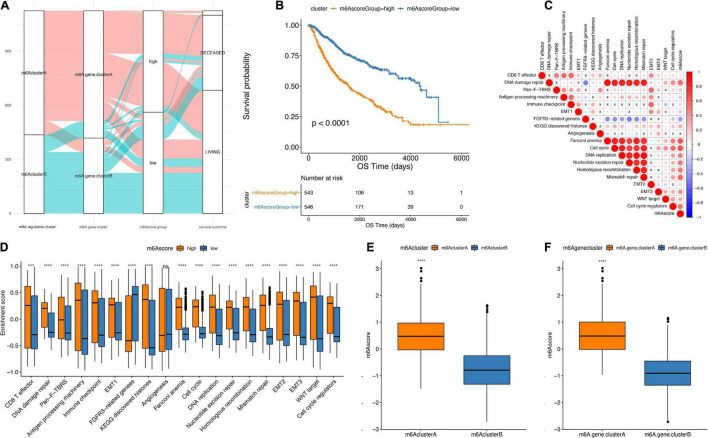
Establishment of m6Ascore. **(A)** Alluvial plot showing the changes of m6A cluster, gene cluster and m6Ascore. **(B)** Kaplan-Meier curve shows that m6Ascore high and low grouping has a significant relationship with overall survival rate. **(C)** Using Pearson analysis, the correlation between m6Ascore and known gene features in low-grade gliomas. Negative correlation is marked in blue, and it is positively correlated with red. X in the figure indicates that the correlation is not significant, and the larger the circle, the more significant. **(D)** The distribution of the enrichment scores of known gene features in the m6Ascore high and low group samples in the TCGA+CGGA data set (***p < 0.01, ****p < 0.001). **(E)** The distribution of m6Ascore in m6A regulators cluster (****p < 0.001). **(F)** Distribution of m6Ascore in m6Agenecluster (****p < 0.001).

The Random Forest algorithm was used to remove the redundancy in the differentially expressed genes, and the characteristic genes most relevant to the classification were identified. A Cox regression model was then used to determine the relationship between these genes and the survival of LGG patients. Based on the coefficient value of the genes, the genes were divided into two categories, and the m6Ascore was calculated in all samples (Figure 6A). Finally, according to the median m6Ascore, the samples were divided into two groups: m6Ascore^high^ and m6Ascore^low^. As presented in Figure 6B, the m6Ascore^low^ group showed significantly better prognosis than m6Ascore^high^ group (*p* < 0.0001), indicating that the calculation based on the m6Ascore provides an accurate characterization of patient prognosis.

The correlation analysis between m6Ascores and known gene features showed that the m6Ascore indicated significantly positive correlation with biological functions, such as DNA damage repair, DNA replication and cell cycle pathways (Figure 6C and Table 3). Importantly, m6Ascore^high^ subgroup was also highly enriched in immune cells infiltrations (CD8 T effector, immune checkpoint, antigen processing machinery), malignant biologic behaviors (EMT process, angiogenesis, WNT targets) and DNA processing (DNA damage repair, DNA replication, homologous recombination, cell cycle regulators, nucleotide excision repair, mismatch repair) (Figure 6D). The Wilcox test showed that m6A regulators clusters and m6Ageneclusters were significantly associated with different m6Ascores (Figures 6E,F). The m6Ascores in m6A regulators clusterA and m6AgeneclusterA were significantly higher than other groups.

**TABLE 3:** Correlation analysis between m6Ascore and known gene characteristics.

	CD8 T effector	DNA damage repair	Pan-F-TBRS	Antigen processing machinery	Immune checkpoint	EMT1	FGFR3-related genes	KEGG discovered histones	Angiogenesis	Fanconi anemia	Cell cycle	DNA replication	Nucleotide excision repair	Homologous recombination	Mismatch repair	EMT2	EMT3	WNT target	Cell cycle regulators	m6Ascore
																				
CD8 T effector	1	–0.06623	0.494146	0.536072	0.598255	0.245074	0.029818	0.240562	0.269904	0.002989	0.082286	0.124355	0.016115	0.063399	0.112345	0.43682	0.198802	–0.11137	0.15242	0.10087
DNA damage repair	–0.06623	1	0.024836	0.116577	–0.13442	0.011223	–0.47825	0.146823	–0.12574	0.878726	0.813629	0.824877	0.836755	0.820814	0.820688	–0.16487	0.126626	0.309855	0.441637	0.584229
Pan-F-TBRS	0.494146	0.024836	1	0.438899	0.34292	0.28936	–0.05985	0.132219	0.406115	0.066112	0.196143	0.173792	0.092248	0.129053	0.174611	0.549272	0.224397	–0.01538	0.184433	0.212418
Antigen processing machinery	0.536072	0.116577	0.438899	1	0.541629	0.187812	–0.16389	0.072601	0.167215	0.064941	0.175006	0.281397	0.277245	0.247106	0.274653	0.415263	0.053926	–0.13306	0.125629	0.203783
Immune checkpoint	0.598255	–0.13442	0.34292	0.541629	1	0.356499	–0.02195	0.269656	0.068756	–0.07124	–0.04095	0.043847	0.022381	0.054325	0.058231	0.572692	0.142592	–0.24812	0.065915	0.176486
EMT1	0.245074	0.011223	0.28936	0.187812	0.356499	1	–0.08486	0.315416	0.016401	0.093048	0.15701	0.110437	0.065016	0.118769	0.119983	0.411339	0.327001	–0.02501	0.108318	0.23616
FGFR3-related genes	0.029818	–0.47825	–0.05985	–0.16389	–0.02195	–0.08486	1	–0.03991	0.132106	–0.41741	–0.40019	–0.37222	–0.48137	–0.43936	–0.40138	0.02771	0.006555	–0.09827	–0.21446	–0.39919
KEGG discovered histones	0.240562	0.146823	0.132219	0.072601	0.269656	0.315416	–0.03991	1	–0.06394	0.250681	0.292092	0.241739	0.119934	0.21112	0.255154	0.262104	0.280397	0.079615	0.237842	0.320571
Angiogenesis	0.269904	–0.12574	0.406115	0.167215	0.068756	0.016401	0.132106	–0.06394	1	–0.14645	–0.01328	–0.05129	–0.11277	–0.15847	–0.07124	0.17815	0.284032	0.036217	0.041086	–0.03072
Fanconi anemia	0.002989	0.878726	0.066112	0.064941	–0.07124	0.093048	–0.41741	0.250681	–0.14645	1	0.833289	0.803697	0.708281	0.847198	0.793349	–0.10502	0.182189	0.282467	0.481197	0.61288
Cell cycle	0.082286	0.813629	0.196143	0.175006	–0.04095	0.15701	–0.40019	0.292092	–0.01328	0.833289	1	0.821026	0.719566	0.751727	0.824546	0.004818	0.339261	0.404269	0.545496	0.660695
DNA replication	0.124355	0.824877	0.173792	0.281397	0.043847	0.110437	–0.37222	0.241739	–0.05129	0.803697	0.821026	1	0.839333	0.868693	0.915835	0.000474	0.168178	0.170521	0.345734	0.481268
Nucleotide excision repair	0.016115	0.836755	0.092248	0.277245	0.022381	0.065016	–0.48137	0.119934	–0.11277	0.708281	0.719566	0.839333	1	0.799102	0.852096	0.00519	0.100386	0.145031	0.294501	0.518847
Homologous recombination	0.063399	0.820814	0.129053	0.247106	0.054325	0.118769	–0.43936	0.21112	–0.15847	0.847198	0.751727	0.868693	0.799102	1	0.84498	0.022523	0.072592	0.123744	0.352855	0.533086
Mismatch repair	0.112345	0.820688	0.174611	0.274653	0.058231	0.119983	–0.40138	0.255154	–0.07124	0.793349	0.824546	0.915835	0.852096	0.84498	1	0.046523	0.196592	0.158169	0.384789	0.595633
EMT2	0.43682	–0.16487	0.549272	0.415263	0.572692	0.411339	0.02771	0.262104	0.17815	–0.10502	0.004818	0.000474	0.00519	0.022523	0.046523	1	0.155017	–0.16645	0.035051	0.174384
EMT3	0.198802	0.126626	0.224397	0.053926	0.142592	0.327001	0.006555	0.280397	0.284032	0.182189	0.339261	0.168178	0.100386	0.072592	0.196592	0.155017	1	0.315586	0.322768	0.408854
WNT target	–0.11137	0.309855	–0.01538	–0.13306	–0.24812	–0.02501	–0.09827	0.079615	0.036217	0.282467	0.404269	0.170521	0.145031	0.123744	0.158169	–0.16645	0.315586	1	0.386595	0.329243
Cell cycle regulators	0.15242	0.441637	0.184433	0.125629	0.065915	0.108318	–0.21446	0.237842	0.041086	0.481197	0.545496	0.345734	0.294501	0.352855	0.384789	0.035051	0.322768	0.386595	1	0.512414
m6Ascore	0.10087	0.584229	0.212418	0.203783	0.176486	0.23616	–0.39919	0.320571	–0.03072	0.61288	0.660695	0.481268	0.518847	0.533086	0.595633	0.174384	0.408854	0.329243	0.512414	1

**FIGURE 7:**
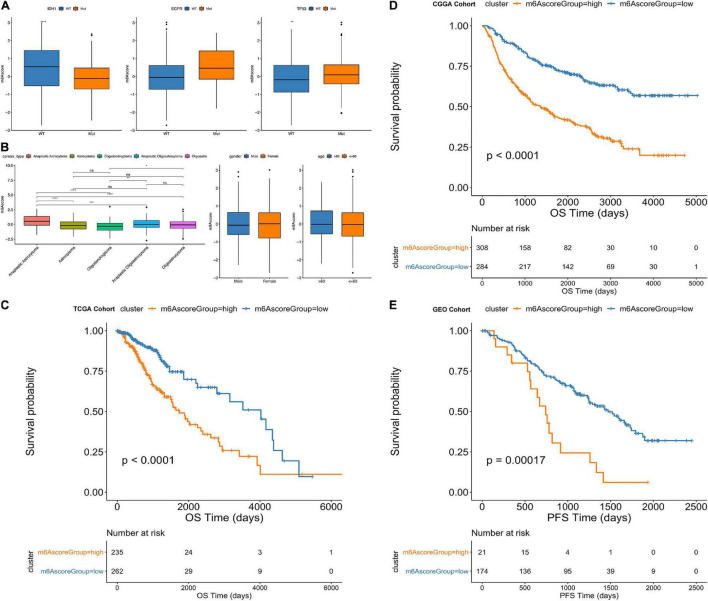
Comparative analysis and model verification of m6Ascore in TCGA dataset. **(A–D)** Distribution of m6Ascore in different classification subgroups. **(A)** IDH1, EGFR, TP53. **(B)** Cancer type. **(C)** Gender. **(D)** Age. **(E)** Difference in survival between m6Ascore high and low groups in the TCGA samples. (*p* < 0.0001).

Furthermore, from the testing TCGA cohort, our analysis revealed that the m6Ascore was significantly different among the classification subgroups (including *IDH1* mutation status, *TP53* mutation status, cancer subtype classification, gender and age) (Figures 7A,B). Additionally, the m6Ascore^high^ subgroup predicts significantly decreased outcomes of LGG compared with m6Ascore^low^ subgroup (*p* < 0.0001; Figure 7C). Then, we chose the CGGA database and GEO database (GSE107850) to verify the survival prediction ability of m6Ascore. We directly extracted the m6Ascore grouping of each sample from the CGGA database, and then plotted the KM curve. It can be seen that the survival results of patients in the m6Ascorehigh subgroup were significantly lower (*p* < 0.0001; Figure 7D). In GSE107850, we selected 195 samples and determined the best classification threshold according to the R function surv_cutpoint (cutoff = 1.233548). Through the KM curve, we found that the m6Ascorehigh subgroup predicted a significant decrease in LGG results (*p* = 0.00017; Figure 7E).

### Differential Molecular Characteristics in m6Ascore^high^ and m6Ascore^low^ Group

**FIGURE 8:**
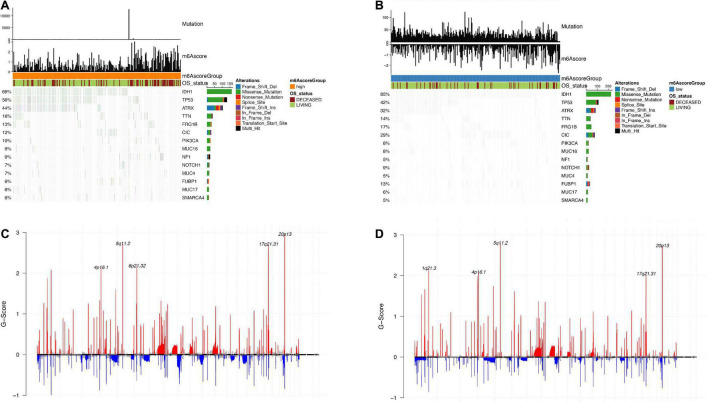
Analysis of molecular characteristics of m6Ascore high and low groups. **(A,B)** Distribution of gene mutations in samples of m6Ascore high and low groups; **(C,D)** The distribution of copy number amplification and deletion regions in the sample set of m6Ascore high and low groups.

Using the TCGA dataset, we further explored the differences between m6Ascore^high^ and m6Ascore^low^ groups. We used “maftools” R package to analyze the differences in somatic mutations between the samples in the m6Ascore^high^ and m6Ascore^low^ groups. As shown in Figures 8A,B, there are significant altered frequency of *IDH1* (69% in m6Ascore^high^, 85% in m6Ascore^low^), *TP53* (55% in m6Ascore^high^, 42% in m6Ascore^low^), *ATRX* (44% in m6Ascore^high^, 32% in m6Ascore^low^), CIC (12% in m6Ascore^high^, 29% in m6Ascore^low^) and FUBP1 (6% in m6Ascore^high^, 13% in m6Ascore^low^) genes. Figures 8C,D show the distribution of copy number variation regions in LGG samples in the m6Ascore^high^ and m6Ascore^low^ groups. In the m6Ascore^high^ group, the deletion regions of CCNA were mainly located in 4p16.1, 5q11.2, 6p21.32, 17q21.3, and 20p13; in the m6Ascore^low^ group, the deletion regions of CCNA were mainly in 1q21.3, 4p16.1, 5q11.2, 17q21.3, and 20p13.

### m6Ascore Predicts Responses to Chemotherapy and Immunotherapy of Low-Grade Glioma

**FIGURE 9:**
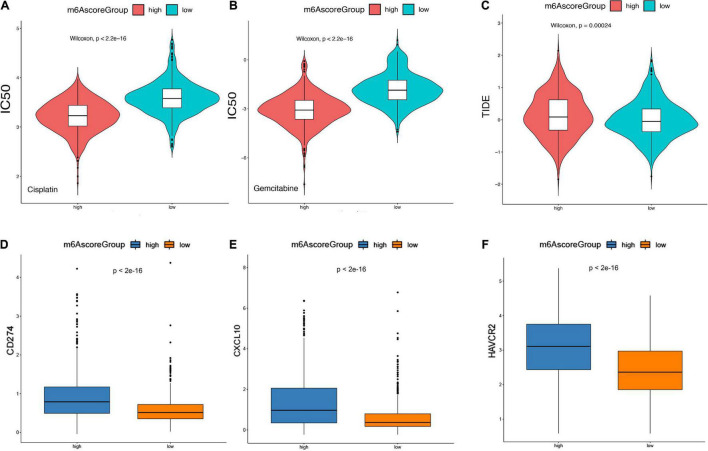
m6Ascore provided predictive outcomes for LGG patients receiving immunotherapies. **(A,B)** The difference between the IC50 values of Cisplatin and Gemcitabine in the samples of the high-risk group and the low-risk group. **(C)** The difference of TIDE score between samples of high-risk group and low-risk group. **(D–F)** Analysis of the degree of correlation between the TIDE score in multiple cohorts and the differential expression of immune checkpoint molecules. **(D)** CD274, **(E)** CXCL10, **(F)** HAVCR2.

Based on 1,586 Chinese and Western LGG patients from TCGA and CGGA database, we used the “pRRophetic” R package to estimate the IC50 value of chemotherapy drugs (cisplatin and gemcitabine) based on the expression profiles and compared the IC50 values of these agents between m6Ascore^high^ and m6Ascore^low^ groups The results showed that the IC50 values in m6Ascore^low^ group was significantly higher than those in m6Ascore^high^ group, indicating that the m6Ascore^high^ patients exhibited poor prognosis and unfavorable responses to chemotherapies (*p* < 2.2e-16; Figures 9A,B).

Furthermore, Tumor Immune Dysfunction and Rejection (TIDE) scores was calculated to evaluate the clinical effects of immune checkpoint inhibitor therapy in m6Ascore^high^ and m6Ascore^low^ groups based on RNA-seq data. As shown in Figure 9C, the TIDE score in m6Ascore^high^ group was significantly higher than m6Ascore^low^ group. In addition, we analyzed the differential expression of immune checkpoint molecules. It suggested that *CD274*, *CXCL19*, and *HAVCR3* expression were significantly increased in m6Ascore^high^ compared with m6Ascore^low^ group (*p* < 0.01; Figures 9D–F). Overall, m6Ascore^high^ brings unfavorable responses for LGG patients received chemotherapies, but preferable responses for LGG patients received immunotherapy, suggesting the determined role of m6Ascore in effective treatment selection.

### Validation of m6A Regulators and Prognostic Role of YTHDF2 in a Real-World Cohort

**FIGURE 10:**
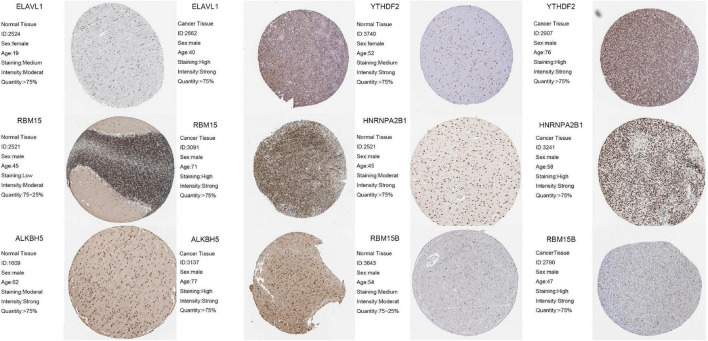
The protein expression of m6A-related genes in normal tissues and tumor tissues.

To further confirm the reliability and prognostic value of m6A-related genes, we selected six m6A regulators, including *ELAVL1*, *YTHDF2*, *RBM15*, *HNRNPA2B1*, *ALKBH5A*, and *RBM15B*, that exhibited the greatest effect on prognosis of LGG. Using immunohistochemistry, we detected protein expression of these genes in normal tissues and tumor tissues. The results showed that ELAVL1, YTHDF2, RBM15, HNRNPA2B1, ALKBH5A, and RBM15B expression was significantly upregulated in tumor tissues compared with normal tissues (*p* < 0.05; Figure 10).

## Discussion

In the past decade, new concepts for the treatment of LGG have emerged, including molecular and genotypic diagnosis, neuroplasticity, function-guided resection and supra-frontal resection (Duffau, 2005; Louis et al., 2016). These ideas have helped improve our understanding of the biological behavior of LGG. However, an important issue that remains to be addressed is that there is no accurate biomarker that can predict the prognosis and deterioration of LGG, which prevents the personalized treatment of these patients (Liu et al., 2020, 2021).

Traditionally, tumor metastasis and invasion were thought to be primarily mediated by genetic and epigenetic variations in tumor cells. Recent research shows that the microenvironment and purity of tumor cells also play a vital role in cancer development (Zhang et al., 2017). Therefore, by comprehensively analyzing the heterogeneity and complexity of the TME, it is possible to identify tumor immunophenotypes, accurate biomarkers, and novel therapeutic targets, thereby improving the ability to predict immunotherapy responses (Yoshihara et al., 2013; Rhee et al., 2018).

As an emerging research direction in oncology, the roles and mechanisms of m6A modification have been investigated by many researchers. Current research suggests that the aberrant expression of m6A regulatory factors is associated with several tumor-related processes, including abnormal cell death, abnormal proliferation, impaired development, tumor invasion, tumor deterioration and immune regulation dysfunction (Han et al., 2019; Wang H. et al., 2019; Zhang et al., 2020). There is an endless stream of research on the role of m6A in gliomas. The latest research combines LGG and GBM to study, and selects genes related to m6A for analysis. The study found that PDPN and TIMP1 can be used as prognostic factors for glioma. Potential biomarkers (Lin et al., 2020). In previous studies, we found that although LGG and GBM are both gliomas, their key markers and TME are not the same. Therefore, in this study, we only chose LGG for analysis. The analysis of GBM will be discussed in the next study. We have also innovatively established m6Ascore to predict the prognosis of LGG patients and the effect of immunotherapy. It is not a single biomarker. This has played a guiding role in revealing the cause of LGG and finding new personalized treatment methods.

Increasing evidence shows that the TME plays an important role in tumor invasion and metastasis. Previous studies have found that the TME-mediated regulation of tumor purity plays a key role in glioma (Fang and Declerck, 2013). Recent research suggests that m6A plays an indispensable role in inflammation, immune environment composition, and tumor progression by interacting with regulatory factors. However, most previous studies analyzed the effect of a single protein on the TME or performed a simple functional analysis of m6A. The investigation of the role of m6A in LGG is even less reported. Therefore, determining the effects of various m6A modification modes on the TME in LGG can improve our understanding of the TME anti-LGG immune response, identify more effective immunotherapy strategies, and lay the foundation for the personalized treatment of LGG patients.

Based on the expression of 21 m6A regulatory factors, we comprehensively evaluated the m6A modification pattern in LGG samples from the TCGA. The expression profiles of 20 m6A genes in LGG samples (no *IGF2BP1* gene expression in the CGGA data) were consistently clustered to obtain m6A regulators clusterA and m6A regulators clusterB. Subsequent analysis of the differences between these groups was performed to obtain m6A-related genes, and then consistent clustering was conducted to obtain m6AgeneclusterA and m6AgeneclusterB. Subsequently, the Random Forest algorithm was used to reduce dimensionality, and Cox regression analysis was performed to identify characteristic genes. We showed that evaluating m6A modification patterns within a single tumor could predict patient prognosis and tumor metastasis. The two clusters were dramatically enriched in different biological processes, specifically cancer-related pathways. We found that m6A regulators clusterA showed a significant immune carcinogenic status, including antigen processing pathways, CD8 T effectors, and immune checkpoints. Based on the infiltration characteristics of TME cells in each m6A regulators cluster, we confirmed that our immunophenotypic classification for different m6A modification patterns correct. Most genes and m6A regulatory factors were overexpressed in m6A regulators clusterA, and the prognosis of m6A regulators clusterA was poor.

Considering that the m6A modification pattern of each patient is unique, we need to quantify the m6A modification mode to enable individualized treatment. To achieve this, we developed an m6A scoring system to analyze the m6A modification pattern in each LGG patient. In our study, we found that the m6Ascores in m6A regulators clusterA and m6AgeneclusterA were significantly higher than those in the other groups, indicating that the m6Ascore can also reflect the TME in the patient. We also observed that the m6Ascore was significantly positively correlated with biological functions, such as DNA replication and cell cycle. Moreover, the m6Ascore exhibited significantly different among various groups of LGG samples depending on *IDH1* mutation, *TP53* mutation status or other LGG subtypes and showed significant association with the prognosis of LGG (Lehrer et al., 2019; Qi et al., 2020), suggesting that the m6Acore is a reliable and valuable tool for comprehensively evaluating the m6A modification pattern in single LGGs, and can be used to conduct a detailed analysis of the LGG immunophenotype in each patient, including the TME status and immune infiltration pattern. Our comprehensive analysis also showed that the m6Acore is an independent prognostic biomarker for LGG. Furthermore, our m6Acore showed a predictive advantage in LGG immunotherapy.

In our study, we found that m6A modification is related to DNA damage repair and DNA replication. Previous studies reported that DNA damage is closely related to autoimmune disorders that trigger inflammatory immune responses. We also found that the m6A modification pattern can affect the components of the LGG TME, such as CD8 T effector cells, or block immune checkpoints to increase treatment resistance (Weenink et al., 2019). Furthermore, a high m6Ascore will promote LGG invasion and infiltration because it may indicate that patients’ angiogenesis, cell cycle changes will aggravate. These factors will likely affect precision immunotherapy in LGG patients. We also found that the m6A modification pattern can shape a variety of substrates and greatly affect the immune TME landscape of LGG. This indicates that m6A modification has an impact on the therapeutic effect of immune checkpoint blockade, highlighting its potential as a new target for immunotherapy. We also confirmed that patients with a high m6Ascore show increased drug resistance to immunotherapy, which may contribute to the variable treatment effects of temozolomide, a classic chemotherapy drug, in different patients. When we evaluated the effect of the TIDE score, the TIDE score in the high m6Ascore group was also higher, indicating that a decreased efficacy of immune checkpoint therapy was associated with a lower survival rate of patients treated with anti-PD1 and anti-CTLA4 therapy.

## Conclusion

In conclusion, this study first demonstrated that m6A modification plays an important role in tumorigenesis and TME infiltration characterization of LGG based on large-scale cohorts. The m6Ascore could accurately predict prognosis and clinical responses to chemotherapy and immunotherapy for LGG patients, which provides novel insights and directions for exploring underlying pathogenesis and identifying novel targets for the treatment of LGG patients.

## Data Availability Statement

The datasets presented in this study can be found in online repositories. The names of the repository/repositories and accession number(s) can be found in the article/Supplementary Material.

## Author Contributions

The work presented here was carried out with the collaboration of all authors. SZ and JW defined the research topic and discussed the analyses and their interpretation, and presentation. WL, HZ, and HH developed the algorithm, found clinicopathological data, and performed the statistical analyses. WL and WX drafted the manuscript, recorded the clinical data, and interpreted the results. CL, YW, and SC participated in reviewing all clinical records and performed the associated data collection. All authors read and approved the final manuscript.

## Conflict of Interest

The authors declare that the research was conducted in the absence of any commercial or financial relationships that could be construed as a potential conflict of interest.

## Publisher’s Note

All claims expressed in this article are solely those of the authors and do not necessarily represent those of their affiliated organizations, or those of the publisher, the editors and the reviewers. Any product that may be evaluated in this article, or claim that may be made by its manufacturer, is not guaranteed or endorsed by the publisher.

## Footnotes

1
https://www.gsea-msigdb.org/gsea/index.jsp

2
https://cloud.genepattern.org/gp/pages/index.jsf

3
http://tide.dfci.harvard.edu/

## Supplementary Material

Supplementary Figure 1 The flowchart of our research.

Supplementary Figure 2 The relationship between m6A regulators and the prognosis of LGG.

## Abbreviations

AUC: area under the curve
CGGA: Chinese Glioma Genome Atlas
CNV: Copy number variation
DEGs: Differential expressed genes
EMT: epithelial-mesenchymal transitions
GGI: Gene-gene interaction
GSVA: Gene set variation analysis
LGG: low grade glioma
OS: overall survival
ROC: Receiver operating characteristic
ssGSEA: single sample Gene Set Enrichment Analysis
TCGA: the Cancer Genome Atlas
TIDE: tumor immune dysfunction and exclusion
TME: tumor microenvironment.
