# Supplementary figures and images for: Integrating m6A Regulators-Mediated Methylation Modification Models and Tumor Immune Microenvironment Characterization in Caucasian and Chinese Low-Grade Gliomas
Source: Front Cell Dev Biol. 2021 Nov 25;9:725764. doi: 10.3389/fcell.2021.725764 (PMC8661096; doi:10.3389/fcell.2021.725764)

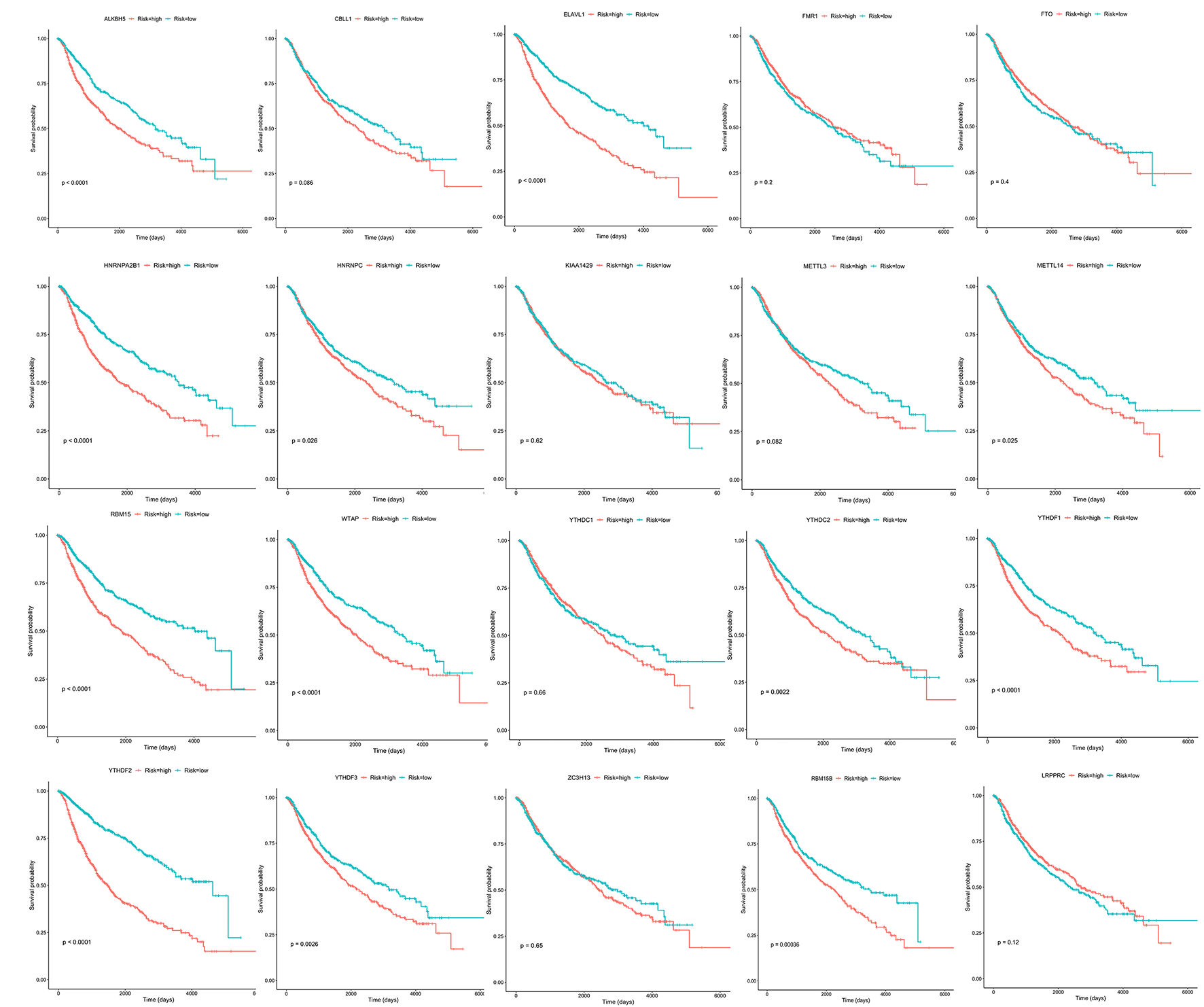

Supplement: Supplementary Figure 1 — The flowchart of our research. [file Image_1.TIF]

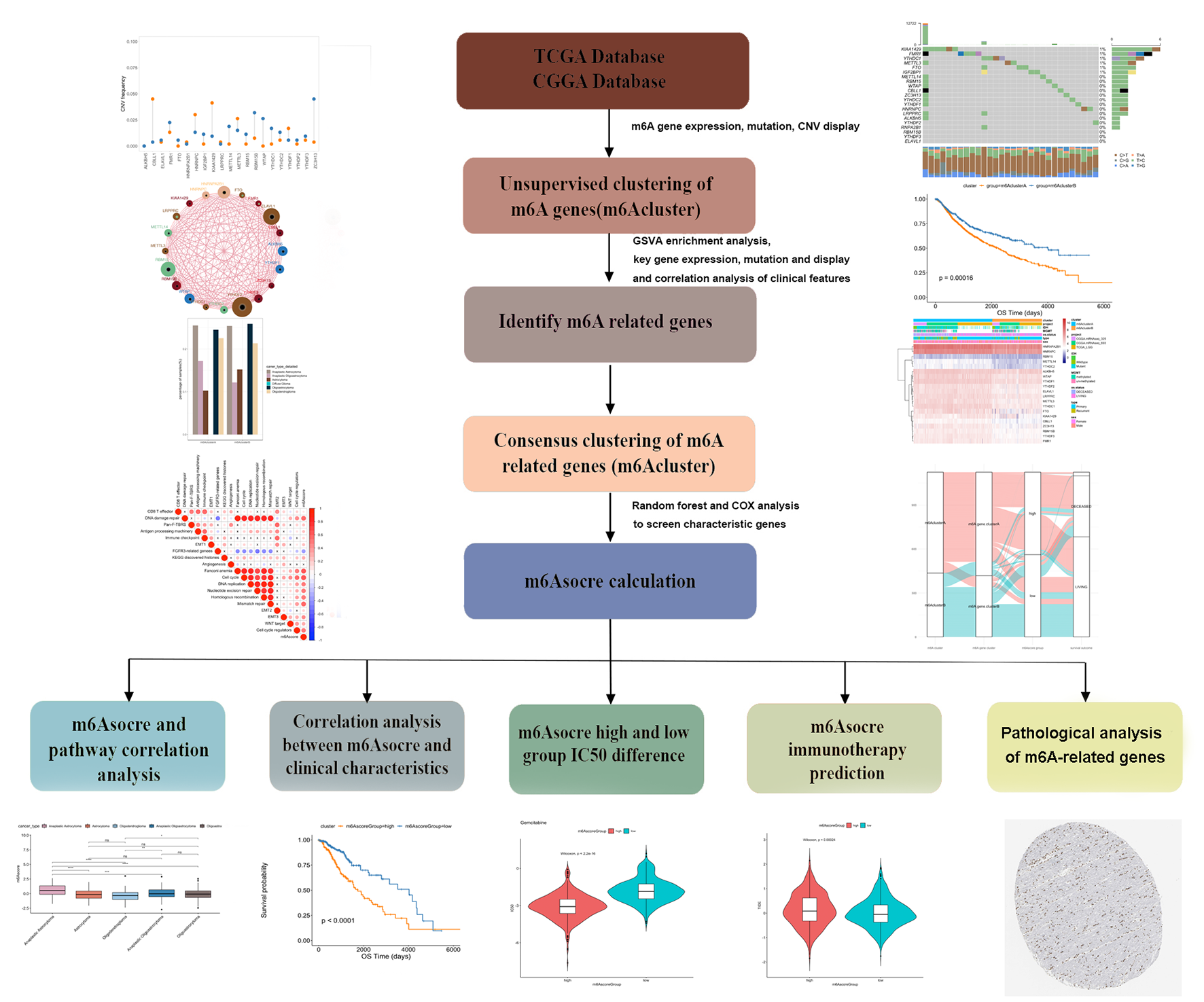

Supplement: Supplementary Figure 2 — The relationship between m6A regulators and the prognosis of LGG. [file Image_2.TIF]
