# Supplementary material for: Integrating m6A Regulators-Mediated Methylation Modification Models and Tumor Immune Microenvironment Characterization in Caucasian and Chinese Low-Grade Gliomas
Source: Front Cell Dev Biol. 2021 Nov 25;9:725764. doi: 10.3389/fcell.2021.725764 (PMC8661096; doi:10.3389/fcell.2021.725764)
Supplement: Supplementary file 3 [file Table_1.docx]

Suppplementary Table1 The interaction of m6A regulatory factors and the connection of regulatory factors

Table 1A Celluar-interactions

| from | to | spearman | pvalue |
| --- | --- | --- | --- |
| ALKBH5 | CBLL1 | 0.160575 | 6.21E-08 |
| ALKBH5 | ELAVL1 | 0.261722 | 4.63E-19 |
| ALKBH5 | FTO | 0.137495 | 3.72E-06 |
| ALKBH5 | HNRNPA2B1 | 0.191752 | 9.07E-11 |
| ALKBH5 | HNRNPC | 0.191485 | 9.63E-11 |
| ALKBH5 | LRPPRC | 0.107879 | 0.000291 |
| ALKBH5 | METTL14 | 0.193625 | 5.90E-11 |
| ALKBH5 | RBM15B | 0.26561 | 1.33E-19 |
| ALKBH5 | WTAP | 0.333689 | 1.23E-30 |
| ALKBH5 | YTHDC1 | 0.141278 | 1.98E-06 |
| ALKBH5 | YTHDC2 | 0.108631 | 0.000264 |
| ALKBH5 | YTHDF1 | 0.312714 | 6.39E-27 |
| ALKBH5 | YTHDF2 | 0.355159 | 9.40E-35 |
| ALKBH5 | YTHDF3 | 0.130379 | 1.16E-05 |
| ALKBH5 | ZC3H13 | 0.151713 | 3.22E-07 |
| CBLL1 | ELAVL1 | 0.625788 | 3.21E-123 |
| CBLL1 | FMR1 | 0.614396 | 1.28E-117 |
| CBLL1 | FTO | 0.48657 | 7.32E-68 |
| CBLL1 | HNRNPA2B1 | 0.499336 | 6.34E-72 |
| CBLL1 | HNRNPC | 0.463434 | 6.34E-61 |
| CBLL1 | KIAA1429 | 0.260074 | 7.82E-19 |
| CBLL1 | LRPPRC | 0.556644 | 1.89E-92 |
| CBLL1 | METTL14 | 0.626289 | 1.79E-123 |
| CBLL1 | METTL3 | 0.413866 | 9.63E-48 |
| CBLL1 | RBM15 | 0.345028 | 9.02E-33 |
| CBLL1 | RBM15B | 0.570843 | 3.53E-98 |
| CBLL1 | WTAP | 0.324806 | 5.00E-29 |
| CBLL1 | YTHDC1 | 0.626044 | 2.38E-123 |
| CBLL1 | YTHDC2 | 0.523823 | 3.24E-80 |
| CBLL1 | YTHDF1 | 0.503407 | 2.95E-73 |
| CBLL1 | YTHDF2 | 0.317836 | 8.42E-28 |
| CBLL1 | YTHDF3 | 0.754381 | 2.45E-207 |
| CBLL1 | ZC3H13 | 0.577355 | 6.69E-101 |
| ELAVL1 | FMR1 | 0.383114 | 1.31E-40 |
| ELAVL1 | FTO | 0.320649 | 2.72E-28 |
| ELAVL1 | HNRNPA2B1 | 0.566089 | 3.14E-96 |
| ELAVL1 | HNRNPC | 0.534835 | 3.60E-84 |
| ELAVL1 | KIAA1429 | 0.282558 | 4.46E-22 |
| ELAVL1 | LRPPRC | 0.329243 | 7.97E-30 |
| ELAVL1 | METTL14 | 0.55772 | 7.11E-93 |
| ELAVL1 | METTL3 | 0.410144 | 7.72E-47 |
| ELAVL1 | RBM15 | 0.466499 | 8.19E-62 |
| ELAVL1 | RBM15B | 0.64895 | 2.34E-135 |
| ELAVL1 | WTAP | 0.512792 | 2.14E-76 |
| ELAVL1 | YTHDC1 | 0.509853 | 2.11E-75 |
| ELAVL1 | YTHDC2 | 0.439214 | 3.28E-54 |
| ELAVL1 | YTHDF1 | 0.564922 | 9.34E-96 |
| ELAVL1 | YTHDF2 | 0.553258 | 4.00E-91 |
| ELAVL1 | YTHDF3 | 0.629013 | 7.54E-125 |
| ELAVL1 | ZC3H13 | 0.35855 | 1.97E-35 |
| FMR1 | FTO | 0.441347 | 8.84E-55 |
| FMR1 | HNRNPA2B1 | 0.389693 | 4.51E-42 |
| FMR1 | HNRNPC | 0.253291 | 6.50E-18 |
| FMR1 | KIAA1429 | 0.108951 | 0.000253 |
| FMR1 | LRPPRC | 0.575427 | 4.34E-100 |
| FMR1 | METTL14 | 0.535888 | 1.48E-84 |
| FMR1 | METTL3 | 0.394497 | 3.67E-43 |
| FMR1 | RBM15 | 0.112304 | 0.000161 |
| FMR1 | RBM15B | 0.322068 | 1.53E-28 |
| FMR1 | WTAP | 0.204061 | 4.97E-12 |
| FMR1 | YTHDC1 | 0.600571 | 4.01E-111 |
| FMR1 | YTHDC2 | 0.607485 | 2.48E-114 |
| FMR1 | YTHDF1 | 0.298519 | 1.44E-24 |
| FMR1 | YTHDF2 | 0.122238 | 3.97E-05 |
| FMR1 | YTHDF3 | 0.600734 | 3.38E-111 |
| FMR1 | ZC3H13 | 0.552398 | 8.65E-91 |
| FTO | HNRNPA2B1 | 0.102772 | 0.000559 |
| FTO | HNRNPC | 0.19647 | 3.04E-11 |
| FTO | KIAA1429 | -0.12599 | 2.27E-05 |
| FTO | LRPPRC | 0.31924 | 4.79E-28 |
| FTO | METTL14 | 0.471768 | 2.31E-63 |
| FTO | METTL3 | 0.209341 | 1.35E-12 |
| FTO | RBM15 | 0.248133 | 3.12E-17 |
| FTO | RBM15B | 0.321607 | 1.84E-28 |
| FTO | YTHDC1 | 0.444223 | 1.48E-55 |
| FTO | YTHDC2 | 0.397401 | 7.88E-44 |
| FTO | YTHDF1 | 0.140207 | 2.37E-06 |
| FTO | YTHDF2 | 0.151266 | 3.49E-07 |
| FTO | YTHDF3 | 0.534252 | 5.87E-84 |
| FTO | ZC3H13 | 0.558231 | 4.47E-93 |
| HNRNPA2B1 | HNRNPC | 0.569985 | 7.97E-98 |
| HNRNPA2B1 | KIAA1429 | 0.292319 | 1.39E-23 |
| HNRNPA2B1 | LRPPRC | 0.200446 | 1.19E-11 |
| HNRNPA2B1 | METTL14 | 0.476292 | 1.03E-64 |
| HNRNPA2B1 | METTL3 | 0.716431 | 1.23E-177 |
| HNRNPA2B1 | RBM15 | 0.275622 | 4.81E-21 |
| HNRNPA2B1 | RBM15B | 0.53401 | 7.20E-84 |
| HNRNPA2B1 | WTAP | 0.521278 | 2.54E-79 |
| HNRNPA2B1 | YTHDC1 | 0.590516 | 1.36E-106 |
| HNRNPA2B1 | YTHDC2 | 0.54932 | 1.34E-89 |
| HNRNPA2B1 | YTHDF1 | 0.607425 | 2.65E-114 |
| HNRNPA2B1 | YTHDF2 | 0.474586 | 3.35E-64 |
| HNRNPA2B1 | YTHDF3 | 0.425562 | 1.17E-50 |
| HNRNPA2B1 | ZC3H13 | 0.29836 | 1.52E-24 |
| HNRNPC | KIAA1429 | 0.435842 | 2.56E-53 |
| HNRNPC | LRPPRC | 0.22867 | 8.43E-15 |
| HNRNPC | METTL14 | 0.420689 | 1.98E-49 |
| HNRNPC | METTL3 | 0.481223 | 3.28E-66 |
| HNRNPC | RBM15 | 0.301788 | 4.24E-25 |
| HNRNPC | RBM15B | 0.568479 | 3.32E-97 |
| HNRNPC | WTAP | 0.441442 | 8.34E-55 |
| HNRNPC | YTHDC1 | 0.552886 | 5.59E-91 |
| HNRNPC | YTHDC2 | 0.212857 | 5.55E-13 |
| HNRNPC | YTHDF1 | 0.450967 | 2.12E-57 |
| HNRNPC | YTHDF2 | 0.514456 | 5.79E-77 |
| HNRNPC | YTHDF3 | 0.428904 | 1.63E-51 |
| HNRNPC | ZC3H13 | 0.271069 | 2.21E-20 |
| KIAA1429 | LRPPRC | 0.285118 | 1.82E-22 |
| KIAA1429 | METTL14 | 0.171856 | 6.69E-09 |
| KIAA1429 | METTL3 | 0.232865 | 2.63E-15 |
| KIAA1429 | RBM15 | 0.096739 | 0.001165 |
| KIAA1429 | RBM15B | 0.290451 | 2.73E-23 |
| KIAA1429 | WTAP | 0.234785 | 1.53E-15 |
| KIAA1429 | YTHDC1 | 0.256281 | 2.58E-18 |
| KIAA1429 | YTHDC2 | 0.08387 | 0.004898 |
| KIAA1429 | YTHDF1 | 0.252909 | 7.31E-18 |
| KIAA1429 | YTHDF2 | 0.278263 | 1.96E-21 |
| KIAA1429 | YTHDF3 | 0.344769 | 1.01E-32 |
| KIAA1429 | ZC3H13 | 0.214458 | 3.68E-13 |
| LRPPRC | METTL14 | 0.372393 | 2.70E-38 |
| LRPPRC | METTL3 | 0.225894 | 1.80E-14 |
| LRPPRC | RBM15B | 0.278672 | 1.70E-21 |
| LRPPRC | WTAP | 0.153011 | 2.54E-07 |
| LRPPRC | YTHDC1 | 0.395236 | 2.48E-43 |
| LRPPRC | YTHDC2 | 0.431974 | 2.63E-52 |
| LRPPRC | YTHDF1 | 0.325052 | 4.52E-29 |
| LRPPRC | YTHDF3 | 0.543201 | 2.83E-87 |
| LRPPRC | ZC3H13 | 0.468369 | 2.33E-62 |
| METTL14 | METTL3 | 0.45443 | 2.30E-58 |
| METTL14 | RBM15 | 0.331274 | 3.40E-30 |
| METTL14 | RBM15B | 0.469714 | 9.36E-63 |
| METTL14 | WTAP | 0.472176 | 1.75E-63 |
| METTL14 | YTHDC1 | 0.762968 | 8.02E-215 |
| METTL14 | YTHDC2 | 0.533643 | 9.80E-84 |
| METTL14 | YTHDF1 | 0.543714 | 1.81E-87 |
| METTL14 | YTHDF2 | 0.291525 | 1.86E-23 |
| METTL14 | YTHDF3 | 0.677243 | 1.06E-151 |
| METTL14 | ZC3H13 | 0.515022 | 3.71E-77 |
| METTL3 | RBM15 | 0.197308 | 2.50E-11 |
| METTL3 | RBM15B | 0.461084 | 3.00E-60 |
| METTL3 | WTAP | 0.34576 | 6.53E-33 |
| METTL3 | YTHDC1 | 0.610972 | 5.57E-116 |
| METTL3 | YTHDC2 | 0.558527 | 3.41E-93 |
| METTL3 | YTHDF1 | 0.489938 | 6.45E-69 |
| METTL3 | YTHDF2 | 0.275491 | 5.03E-21 |
| METTL3 | YTHDF3 | 0.362304 | 3.40E-36 |
| METTL3 | ZC3H13 | 0.293099 | 1.05E-23 |
| RBM15 | RBM15B | 0.344446 | 1.17E-32 |
| RBM15 | WTAP | 0.295303 | 4.70E-24 |
| RBM15 | YTHDC1 | 0.293441 | 9.28E-24 |
| RBM15 | YTHDC2 | 0.282619 | 4.36E-22 |
| RBM15 | YTHDF1 | 0.21504 | 3.17E-13 |
| RBM15 | YTHDF2 | 0.555754 | 4.23E-92 |
| RBM15 | YTHDF3 | 0.434791 | 4.84E-53 |
| RBM15 | ZC3H13 | 0.085969 | 0.003922 |
| RBM15B | WTAP | 0.402262 | 5.81E-45 |
| RBM15B | YTHDC1 | 0.534229 | 5.99E-84 |
| RBM15B | YTHDC2 | 0.344854 | 9.75E-33 |
| RBM15B | YTHDF1 | 0.524718 | 1.57E-80 |
| RBM15B | YTHDF2 | 0.504013 | 1.86E-73 |
| RBM15B | YTHDF3 | 0.525358 | 9.30E-81 |
| RBM15B | ZC3H13 | 0.349375 | 1.30E-33 |
| WTAP | YTHDC1 | 0.40001 | 1.95E-44 |
| WTAP | YTHDC2 | 0.320224 | 3.23E-28 |
| WTAP | YTHDF1 | 0.563896 | 2.43E-95 |
| WTAP | YTHDF2 | 0.518741 | 1.94E-78 |
| WTAP | YTHDF3 | 0.435005 | 4.25E-53 |
| WTAP | ZC3H13 | 0.17198 | 6.53E-09 |
| YTHDC1 | YTHDC2 | 0.520341 | 5.39E-79 |
| YTHDC1 | YTHDF1 | 0.499375 | 6.16E-72 |
| YTHDC1 | YTHDF2 | 0.313762 | 4.24E-27 |
| YTHDC1 | YTHDF3 | 0.605395 | 2.36E-113 |
| YTHDC1 | ZC3H13 | 0.544676 | 7.86E-88 |
| YTHDC2 | YTHDF1 | 0.438963 | 3.83E-54 |
| YTHDC2 | YTHDF2 | 0.275225 | 5.50E-21 |
| YTHDC2 | YTHDF3 | 0.590201 | 1.87E-106 |
| YTHDC2 | ZC3H13 | 0.498391 | 1.28E-71 |
| YTHDF1 | YTHDF2 | 0.313702 | 4.34E-27 |
| YTHDF1 | YTHDF3 | 0.474673 | 3.15E-64 |
| YTHDF1 | ZC3H13 | 0.28255 | 4.47E-22 |
| YTHDF2 | YTHDF3 | 0.420251 | 2.55E-49 |
| YTHDF2 | ZC3H13 | 0.154951 | 1.78E-07 |
| YTHDF3 | ZC3H13 | 0.615582 | 3.42E-118 |

Table 1B Celluar-types

|  | hr | lower.95 | upper.95 | pvalue |
| --- | --- | --- | --- | --- |
| ALKBH5 | 1.67815 | 1.380628 | 2.039787 | 1.07E-07 |
| WTAP | 1.493609 | 1.296218 | 1.721059 | 4.86E-08 |
| YTHDF1 | 1.536051 | 1.267215 | 1.86192 | 9.14E-06 |
| CBLL1 | 1.27222 | 1.069882 | 1.512823 | 0.006492 |
| FMR1 | 0.921669 | 0.769999 | 1.103213 | 0.37583 |
| KIAA1429 | 1.13197 | 1.002262 | 1.278465 | 0.044803 |
| RBM15B | 1.574182 | 1.31089 | 1.890356 | 1.58E-06 |
| YTHDF3 | 1.48474 | 1.2277 | 1.795597 | 4.13E-05 |
| ZC3H13 | 1.052246 | 0.911436 | 1.21481 | 0.486037 |
| ELAVL1 | 2.163312 | 1.833817 | 2.552009 | 1.25E-19 |
| FTO | 0.975738 | 0.888133 | 1.071985 | 0.610196 |
| LRPPRC | 0.776445 | 0.662665 | 0.909762 | 0.002095 |
| METTL3 | 1.143254 | 0.988096 | 1.322776 | 0.070012 |
| YTHDC1 | 1.074835 | 0.906737 | 1.274097 | 0.404237 |
| YTHDF2 | 2.555007 | 2.129972 | 3.064858 | 1.28E-25 |
| HNRNPA2B1 | 1.71795 | 1.476455 | 1.998945 | 1.92E-12 |
| HNRNPC | 1.295678 | 1.106794 | 1.516796 | 0.000955 |
| METTL14 | 1.339952 | 1.129086 | 1.590199 | 0.000722 |
| RBM15 | 2.370612 | 1.960012 | 2.867228 | 2.63E-16 |
| YTHDC2 | 1.344173 | 1.140276 | 1.58453 | 0.0004 |
