# Supplementary material for: Integrating m6A Regulators-Mediated Methylation Modification Models and Tumor Immune Microenvironment Characterization in Caucasian and Chinese Low-Grade Gliomas
Source: Front Cell Dev Biol. 2021 Nov 25;9:725764. doi: 10.3389/fcell.2021.725764 (PMC8661096; doi:10.3389/fcell.2021.725764)
Supplement: Supplementary file 4 [file Data_Sheet_1.ZIP › 244673.zip!/244673/high.del_qplot.pdf]

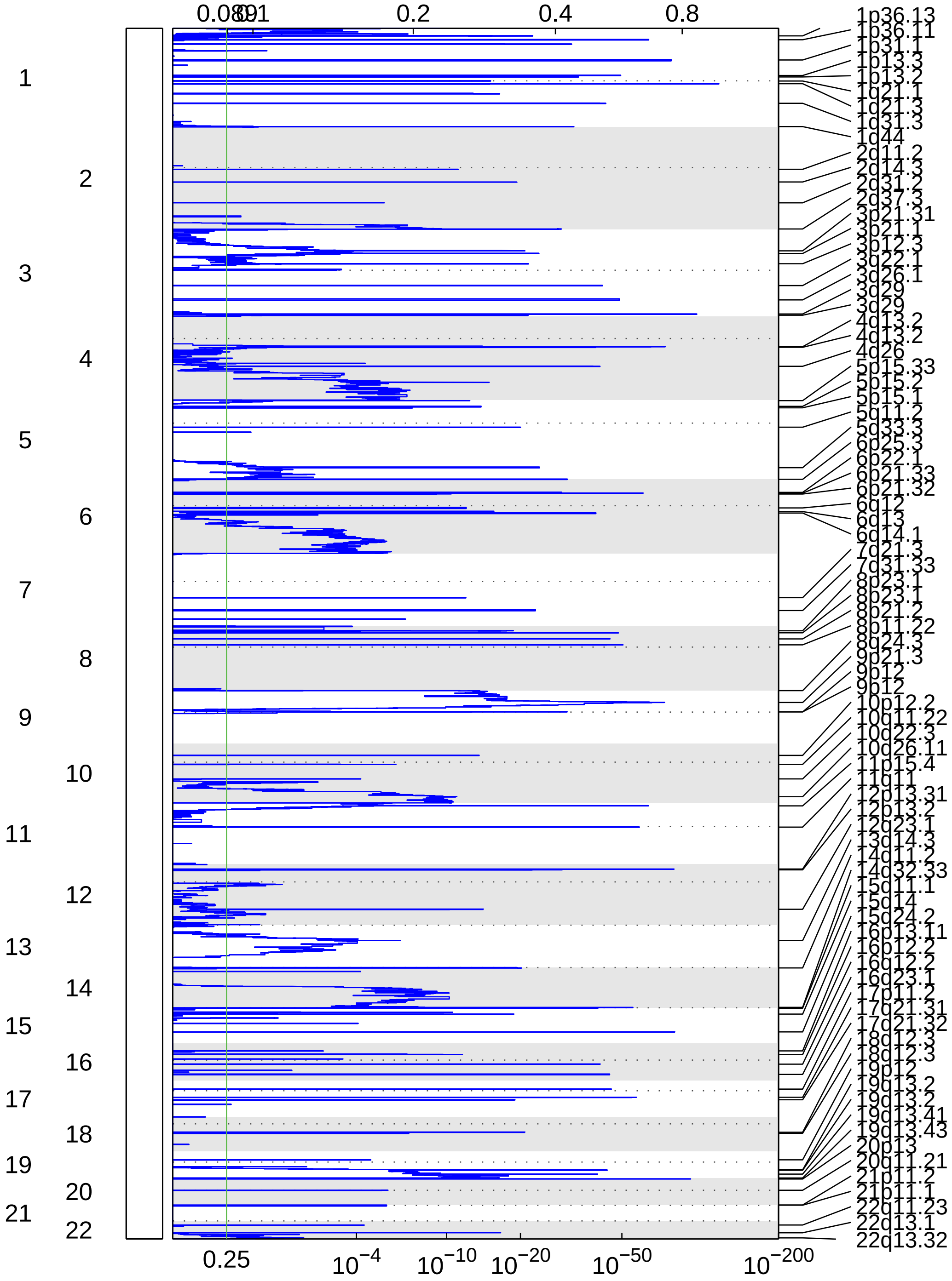
0.08
0.1
0.2
0.4
0.8
1
2
3
4
5
6
7
8
9
10
11
12
13
14
15
16
17
18
19
20
21
22
0.25
10−4
10−10
10−20
10−50
10−200
1p36.13
1p36.11
1p31.1
1p13.3
1p13.2
1q21.1
1q21.3
1q31.3
1q44
2q11.2
2q14.3
2q31.2
2q37.3
3p21.31
3p21.1
3p12.3
3q22.1
3q26.1
3q29
3q29
4q13.2
4q13.2
4q26
5p15.33
5p15.2
5p15.1
5q11.2
5q33.3
6p25.3
6p22.1
6p21.33
6p21.32
6q12
6q13
6q14.1
7q21.3
7q31.33
8p23.1
8p23.1
8p21.2
8p11.22
8q24.3
9p21.3
9p12
9p12
10p12.2
10q11.22
10q22.3
10q26.11
11p15.4
11q11
12p13.31
12p13.2
12q23.1
13q14.3
14q11.2
14q32.33
15q11.1
15q14
15q24.2
16p13.11
16p12.2
16q12.2
16q23.1
17p11.2
17q21.31
17q21.32
18q12.3
18q12.3
19p12
19q13.2
19q13.2
19q13.41
19q13.43
20p13
20q11.21
21p11.2
21p11.1
22q11.23
22q13.1
22q13.32
