# Supplementary material for: Integrating m6A Regulators-Mediated Methylation Modification Models and Tumor Immune Microenvironment Characterization in Caucasian and Chinese Low-Grade Gliomas
Source: Front Cell Dev Biol. 2021 Nov 25;9:725764. doi: 10.3389/fcell.2021.725764 (PMC8661096; doi:10.3389/fcell.2021.725764)
Supplement: Supplementary file 4 [file Data_Sheet_1.ZIP › 244673.zip!/244673/high.freqarms_vs_ngenes.pdf]

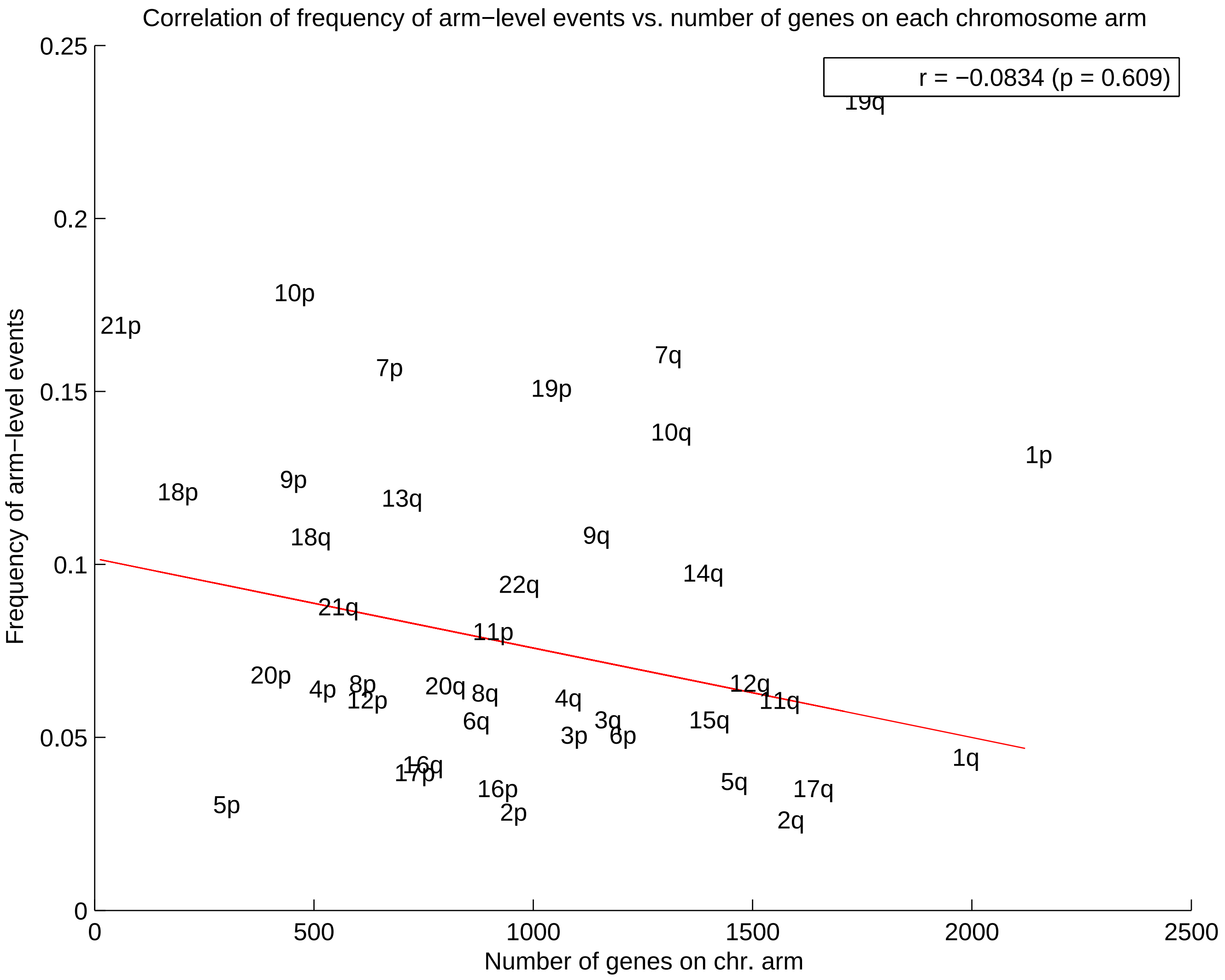
Correlation of frequency of arm−level events vs. number of genes on each chromosome arm
r = −0.0834 (p = 0.609)
Frequency of arm−level events
Number of genes on chr. arm
0.25
0.2
0.15
0.1
0.05
0
0
500
1000
1500
2000
2500
19q
10p
21p
7q
7p
19p
10q
1p
18p
9p
13q
18q
9q
14q
22q
21q
11p
20p
4p
8p
12p
20q
8q
4q
12q
11q
6q
3q
15q
3p
6p
1q
16q
17p
16p
5q
17q
5p
2p
2q
