# Supplementary material for: Integrating m6A Regulators-Mediated Methylation Modification Models and Tumor Immune Microenvironment Characterization in Caucasian and Chinese Low-Grade Gliomas
Source: Front Cell Dev Biol. 2021 Nov 25;9:725764. doi: 10.3389/fcell.2021.725764 (PMC8661096; doi:10.3389/fcell.2021.725764)
Supplement: Supplementary file 4 [file Data_Sheet_1.ZIP › 244675.zip!/244675/low.amp_qplot.pdf]

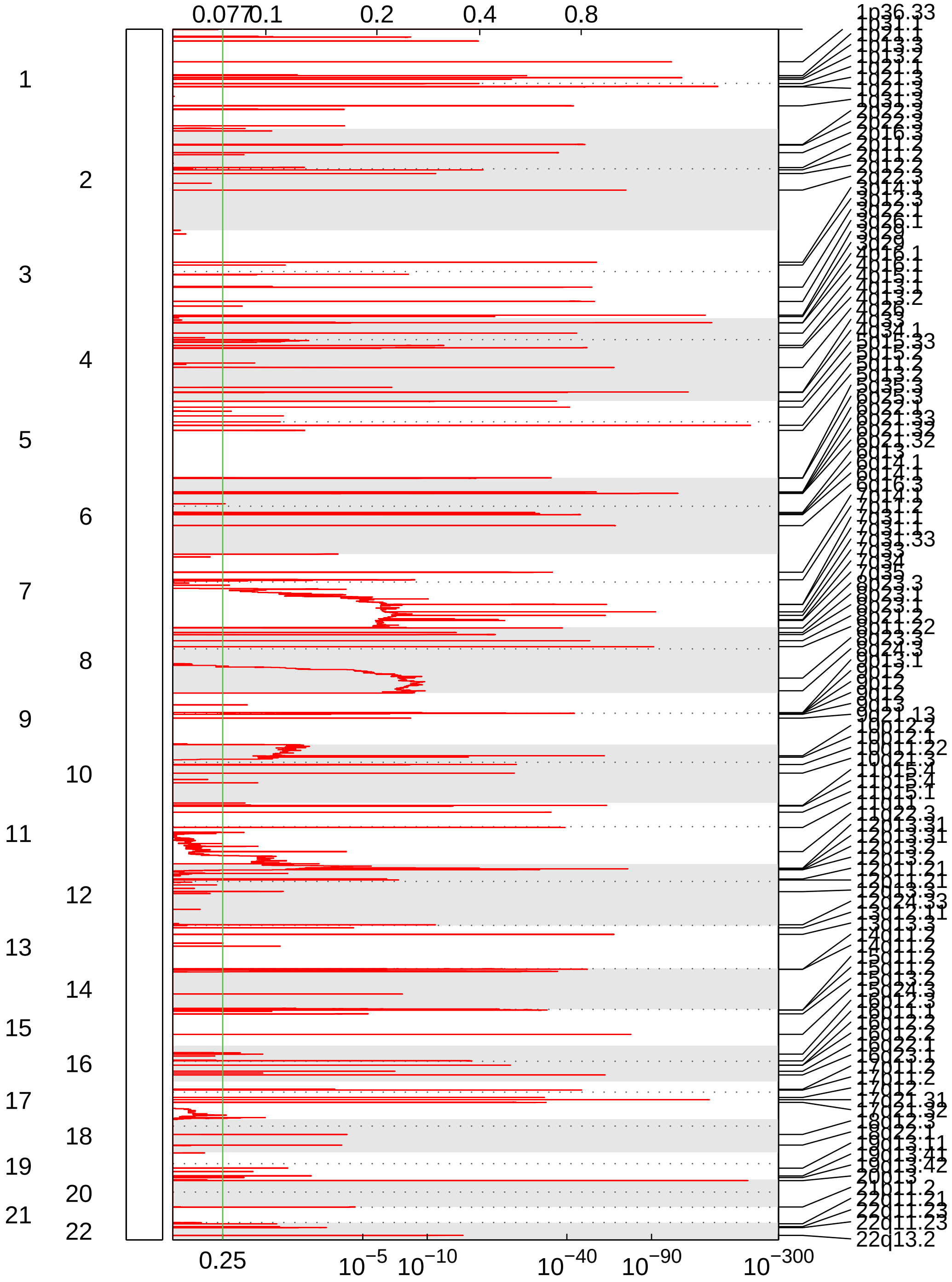
0.077
0.1
0.2
0.4
0.8
1
2
3
4
5
6
7
8
9
10
11
12
13
14
15
16
17
18
19
20
21
22
0.25
10^−5
10^−10
10^−40
10^−90
10^−300
1p36.33
22q13.2
