# Supplementary material for: Integrating m6A Regulators-Mediated Methylation Modification Models and Tumor Immune Microenvironment Characterization in Caucasian and Chinese Low-Grade Gliomas
Source: Front Cell Dev Biol. 2021 Nov 25;9:725764. doi: 10.3389/fcell.2021.725764 (PMC8661096; doi:10.3389/fcell.2021.725764)
Supplement: Supplementary file 4 [file Data_Sheet_1.ZIP › 244675.zip!/244675/low.freqarms_vs_ngenes.pdf]

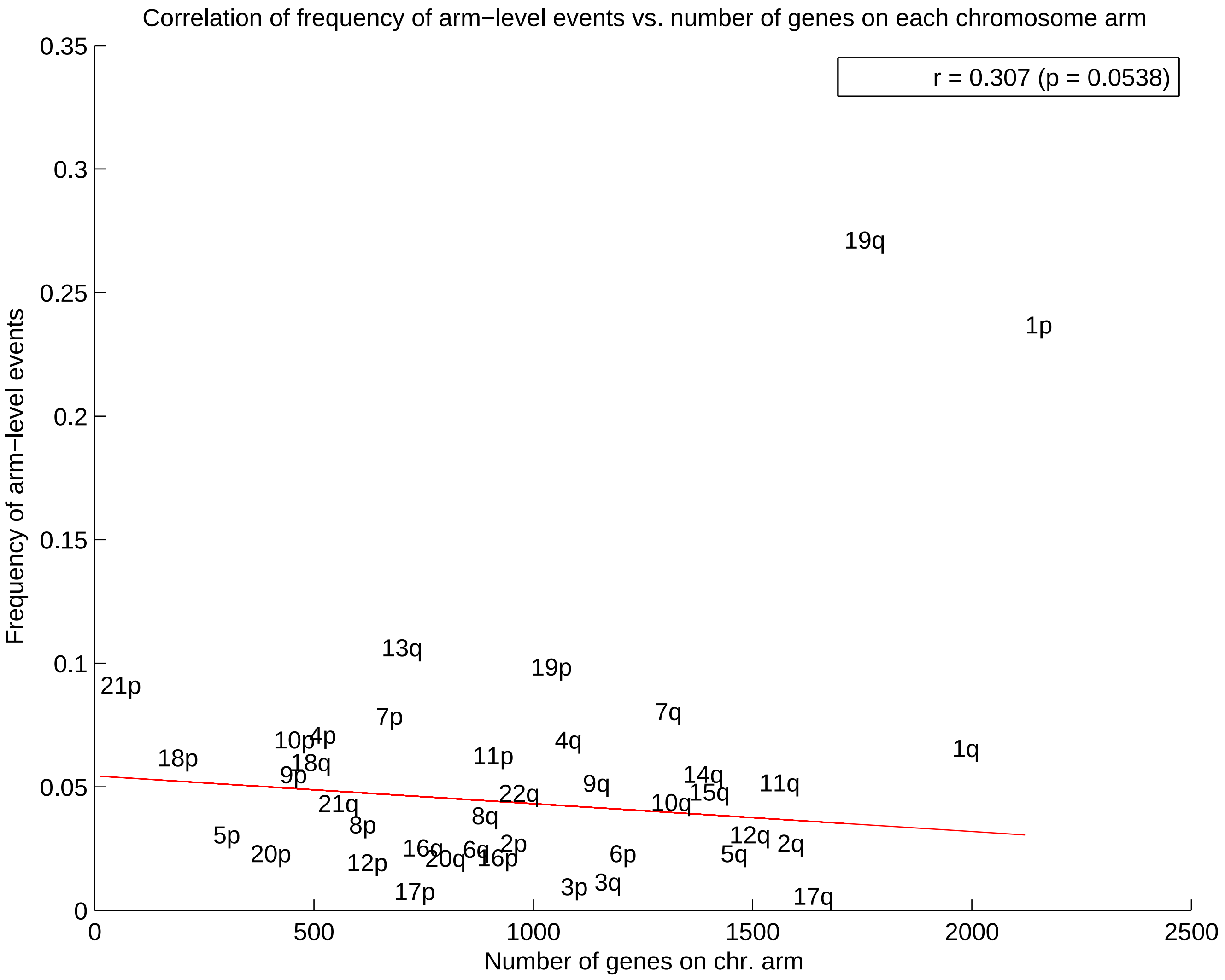

Correlation of frequency of arm−level events vs. number of genes on each chromosome arm
r = 0.307 (p = 0.0538)
Frequency of arm−level events
Number of genes on chr. arm
0
0.05
0.1
0.15
0.2
0.25
0.3
0.35
0
500
1000
1500
2000
2500
19q
1p
13q
19p
21p
7p
7q
10p
4p
4q
18p
18q
11p
1q
9p
14q
9q
11q
22q
15q
21q
10q
8q
8p
5p
12q
2q
20p
16q
6q
2p
16p
20q
6p
5q
12p
3p
3q
17p
17q
